# Third instar larvae of flesh flies (Diptera: Sarcophagidae) of forensic importance—critical review of characters and key for European species

**DOI:** 10.1007/s00436-015-4421-3

**Published:** 2015-04-01

**Authors:** Krzysztof Szpila, René Richet, Thomas Pape

**Affiliations:** 1 Chair of Ecology and Biogeography, Faculty of Biology and Environmental Protection, Nicolaus Copernicus University, Lwowska 1, 87-100 Toruń, Poland; 2 16 Grande Rue, 03220 Jaligny-sur-Besbre, France; 3 Natural History Museum of Denmark, University of Copenhagen, Universitetsparken 15, 2100 Copenhagen, Denmark

**Keywords:** Sarcophagidae, Larva, Key, Species identification, Europe, Forensic entomology

## Abstract

Necrophagous Sarcophagidae are among the insects most frequently reported from human corpses. The broad forensic application of flesh flies is restricted by the lack of reliable tools for species identification of larval stages and mass breeding of collected flesh fly larvae to the adult stage, and more recently DNA-based methods are usually recommended for precise species identification. To overcome this situation, the following study was implemented: (1) original larval material was obtained of the European flesh flies of confirmed or potential forensic importance; (2) larval morphology was studied and documented using a combination of standard light microscopy, image-stacking stereomicroscopy and SEM; and (3) larval characters used in previously published keys were critically revised. The taxonomic value of the following characters was considered insignificant: (1) differences in level of sclerotisation of particular parts of the cephaloskeleton, (2) level of sclerotisation of the posterior spiracular peritreme and (3) the shape of posterior spiracular slits. A high taxonomic value was noticed for the general shape of anterior spiracles, pattern of arrangement of their lobes, and distribution and shape of spines/warts on the inter-band area of segments. Two character states—long window in the dorsal cornu of cephaloskeleton and deep spiracular cavity on anal division—are not found in the Miltogramminae and therefore cannot be considered as family-specific for the entire Sarcophagidae. As a comprehensive result of our studies, an identification key is presented for the third instar larvae of European flesh flies of forensic importance. The key is user-friendly and requires no dissections of larvae, as soaking the material in methyl salicylate will allow observation of all diagnostic details of the cephaloskeleton. A simple stereomicroscope (magnification about ×50) is sufficient for the observation of all characters presented in the key. This key may be systematically extended by the addition of species present in adjacent geographical regions.

## Introduction

Flesh flies in the temperate climate zone are described as a scarce but constant component of carrion communities (Denno and Cothran 1976; Hanski 1987). Larvae of Sarcophagidae can feed on both small and large carrion, including human bodies, and the taxon therefore has considerable forensic importance (Smith 1986; Byrd and Castner 2009). However, the number of flesh fly species which is attracted to large carrion and can develop in this breeding medium is low in relation to the overall diversity of this taxon. In Europe, large carrion is known to attract 33 species of Sarcophagidae (Arnaldos et al. 2004; Grassberger and Frank 2004; Matuszewski et al. 2008; Bonacci et al. 2010; Prado e Castro et al. 2010; Anton et al. 2011). Successional studies and published descriptions of case reports with reliable species identification bring information about only seven species recorded on this kind of substrate as larvae (Povolný and Verves 1997; Benecke 1998; Draber-Mońko et al. 2009; Pohjoismäki et al. 2010; Velásquez et al. 2010; Cherix et al. 2012; Bonacci et al. 2014; Szpila et al. 2015). In Europe, the flesh fly most frequently collected as larva on human corpses is *Sarcophaga argyrostoma* (Povolný and Verves 1997; Benecke 1998; Draber-Mońko et al. 2009; Velásquez et al. 2010; Cherix et al. 2012). Other species like *Sarcophaga caerulescens* and *Sarcophaga similis* are recorded incidentally (Pohjoismäki et al. 2010; Cherix et al. 2012). The remaining three species, *Ravinia pernix*, *Sarcophaga crassipalpis*, and *Sarcophaga cultellata* have been reported from human corpses very rarely and only in Mediterranean countries (Velásquez et al. 2010; Bonacci et al. 2014).

Large-scale studies of ecology of carrion-breeding flesh flies and practical forensic application of this taxon are complicated by serious problems with species identification of larvae. Larval stages of the most common carrion-breeding European flesh flies have been described in numerous papers, but a complete key for their identification does not exist. In this situation, as is the case also for other ecozones, forensic entomology manuals recommend the mass rearing of the collected flesh fly larvae to adults for a precise species identification (Smith 1986; Byrd and Castner 2009). A few existing keys for species identification of larvae of necrophagous Sarcophagidae are restricted to local faunas of geographical regions far from Europe (Greene 1925; Zimin 1948; Bohart and Gressitt 1951; Kano et al. 1951; Sanjean 1957; Ishijima 1967; Nandi 1980; Sukontason et al. 2010) or, if referring to the European fauna, they cover narrow subsets of species (Velásquez et al. 2010; Ubero-Pascal et al. 2015). The recent monograph of Richet et al. (2011) and the molecular work of Jordaens et al. (2013) opened the possibility to overcome the problem of species identification of larvae of most species of European necrophagous flesh flies. However, the molecular methods proposed by Jordaens et al. (2013) are inconvenient for application when a large material is studied, like in ecological experiments. Richet et al. (2011) provide data about larval morphology of large subsets of species, but the information is restricted to photographs of a few selected details of larval morphology and without supporting descriptions or any attempt to construct a key.

The last two decades have brought information which makes it possible to circumscribe a guild of macro-necrophagous Sarcophagidae with a trophic relation to large carrion, including human bodies, at least for Central and North Europe (Povolný and Verves 1997; Benecke 1998; Draber-Mońko et al. 2009; Pohjoismäki et al. 2010; Velásquez et al. 2010; Cherix et al. 2012; Bonacci et al. 2014; Szpila et al. 2015). Based on original larval material and a broad approach to morphological characters, we present an easily applicable key for species identification of larvae of necrophagous flesh flies. The present key is possible to use without dissection of larvae and will be helpful in work with material collected during studies of carrion communities and true forensic cases. The coverage of species is restricted to Central and North Europe, but the key can easily be augmented for application in countries of the Mediterranean and Middle East along with the accumulation of data for larval morphology of species representing these regions.

## Material and methods

Gravid females attracted to decomposing pig or chicken liver were collected by sweep net. Detailed data concerning collecting locations are presented in Table 1. The abdomen of live females was subsequently gently squeezed for extraction of the first instar larvae, which were reared to the third instar in small plastic containers (150 ml). The bottom of each container was covered by a thin layer (2 cm) of humid sand, on the surface of which was placed a small portion of pig or chicken liver (20–30 g). Ten larvae from each female were reared to get adult male specimens for unambiguous species identification. Microscope slides of larvae of *R. pernix* collected in France were provided by the second author. Voucher specimens are available from the Chair of Ecology and Biogeography, NCU.

**Table 1:** Females of Sarcophagidae obtained and their localities

Species “specimen code”	Location	Coordinate	Habitat
*P. pictipennis* “6.06.2010”	Toruń, lotnisko Poland	53° 01′ N, 18° 33′ E	Midland dunes with sparse pine stands
*P. pictipennis* “breeding exp.”	Toruń, lotnisko Poland	53° 01′ N, 18° 33′ E	Midland dunes with sparse pine stands
*S. latifrons* “7.07.2013”	Toruń, lotnisko Poland	53° 01′ N, 18° 33′ E	Midland dunes with sparse pine stands
*S. latifrons* “Sarc 3 Stawki”	Toruń, Stawki Poland	52° 59′ N, 18° 39′ E	Midland dunes with sparse pine stands
*W. trina* “Wohlfahrtia 4 EAkev”	Ein Avdat NP Israel	30° 48′ N, 34° 48′ E	Sparse vegetation along dry stream bed
*W. villeneuvi* “Wohlfahrtia 2 EAkev”	Ein Avdat NP Israel	30° 48′ N, 34° 48′ E	Sparse vegetation along dry stream bed
*S. aegyptica* “Iran Sarc 1”	Marghzār Iran	37° 03′ N, 56° 16′ E	Sparse vegetation along dry stream bed
*S. africa* “garaż”	Toruń Poland	53° 01′ N, 18° 34′ E	Anthropogenic habitat with sparse ruderal vegetation
*S. albiceps* “Sarc 2 Tpol”	Toruń, Góra Żymierskiego Poland	52° 57′ N, 18° 34′ E	Birch forest in early stage of succession
*S. albiceps* “Sarc Dunajec 1”	Wojnicz Poland	49° 57′ N, 20° 52′ E	Meadow at river bank
*S. argyrostoma* “Sarc Tinst 1”	Toruń, Instytut Poland	53° 01′ N, 18° 34′ E	Indoor
*S. argyrostoma* “Sarc Tinst 2”	Toruń, Instytut Poland	53° 01′ N, 18° 34′ E	Indoor
*S. caerulescens* “Sarc 3 ZP”	Zbocza Płutowskie reserve Poland	53° 16′ N, 18° 23′ E	Ecotone between wet meadow/deciduous forest
*S. caerulescens* “Sarc 8 Tlot”	Toruń, lotnisko Poland	53° 01′ N, 18° 33′ E	Midland dunes with sparse pine stands
*S. melanura* “Sarc 10 Tlot”	Toruń, lotnisko Poland	53° 01′ N, 18° 33′ E	Midland dunes with sparse pine stands
*S. melanura* “Sarc 1 Tpol”	Toruń, Góra Żymierskiego Poland	52° 57′ N, 18° 34′ E	Birch forest in early stage of succession
*S. similis* “Sarc 4 Tlot”	Toruń, lotnisko Poland	53° 01′ N, 18° 33′ E	Midland dunes with sparse pine stands
*S. variegata* “11.VIII.2008”	Toruń, lotnisko Poland	53° 01′ N, 18° 33′ E	Hornbeam-oak forest

For all species, live larvae were rinsed in water with a small amount of detergent and subsequently killed by being immersed for a few minutes in hot water (about 95 °C) to extend the pseudocephalon and avoid subsequent deformation when stored in 70 % alcohol.

All ten larvae from each batch were analysed with a M205C Leica Stereomicroscope. The preparation of image stacking was done using the same stereomicroscope with an integrated high-resolution Leica DFC495 digital camera and associated software (Leica Application Suite 4.4.0). For better visibility of spinulation and integumental sculpture, larvae were painted by careful strokes with a blue STABILO marker. Next, larvae were macerated for 24 h in a cold solution of 5 % KOH. Subsequently, particular fragments of the body were dissected and dehydrated through 80, 90 and 99.5 % ethanol and mounted in Euparal. Cephaloskeletons were split into their two symmetric parts by sagittal cutting, and each part was mounted on a flat slide. At least one intact larva of each species was immersed in methyl salicylate for non-invasive observation of the cephaloskeleton (Niederegger et al. 2011). Another ten larvae from a single female were prepared for SEM by dehydration through 80, 90 and 99.5 % ethanol, critical point dried in CO_2_, and sputter-coated with platinum. SEM images were taken with a JEOL JSM-6335 F scanning electron microscope (JEOL Ltd., Tokyo, Japan).

A number of microscope slides of third instar larvae of *Sarcophaga* spp. prepared for the monograph by Richet et al. (2011) were used for comparative studies (*Sarcophaga aegyptica*, *S. africa*, *S. albiceps*, *S. argyrostoma*, *S. caerulescens*, *S. carnaria* species group, *S. dux*, *S. melanura*, *S. similis*, *S. tibialis*).

**Fig. 1:**
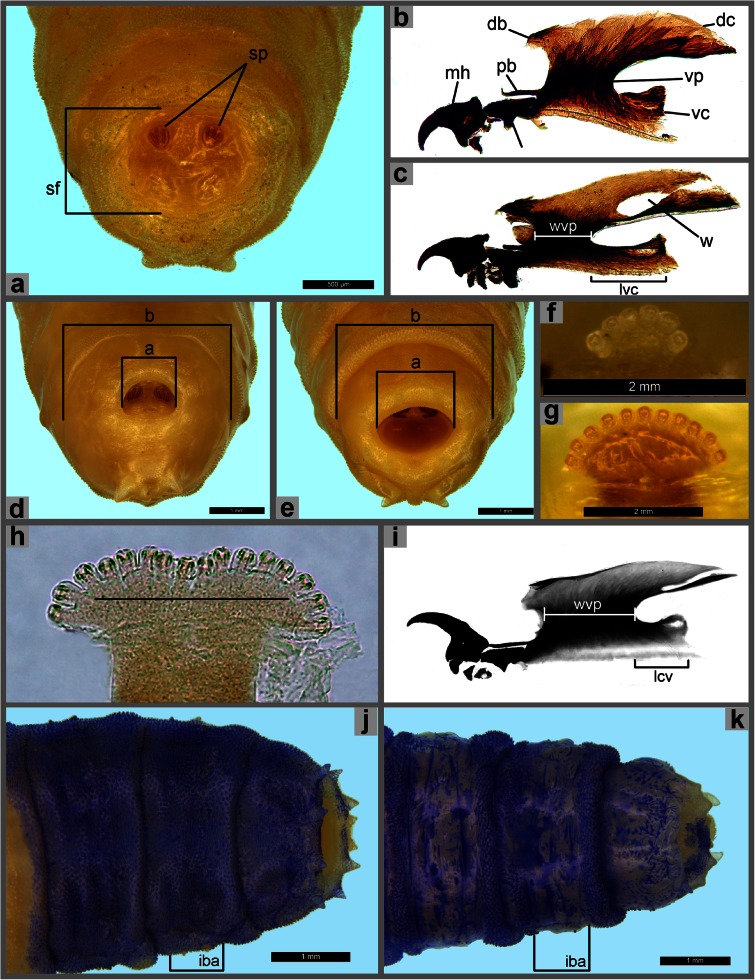
Third instar morphology of Sarcophagidae. **a** Anal division, posterior view, *Phylloteles pictipennis*. **b** Cephaloskeleton, lateral view, *P. pictipennis*. **c** Cephaloskeleton, lateral view, *Sarcophaga argyrostoma*. **d** Anal division, posterior view, *Wohlfahrtia villeneuvi*. **e** Anal division, posterior view, *S. argyrostoma*. **f** Anterior spiracle, *Sarcophila latifrons*. **g** Anterior spiracle, *W. villeneuvi*. **h** Anterior spiracle, *R. pernix*. **i** Cephaloskeleton, lateral view, *R. pernix*. **j** Posterior end of body, dorsal view, *S. melanura*. **j** Posterior end of body, dorsal view, *S. aegyptica*. Abbreviations: *a* width of entrance of spiracular cavity, *b* width of anal division, *db* dorsal bridge, *dc* dorsal cornu, *iba* inter-band area, *is* intermediate sclerite, *lvc* length of ventral cornu, *mh* mouthhooks, *pb* parastomal bar, *sf* spiracular field, *sp* posterior spiracles, *vc* ventral cornu, *vp* vertical plate, *w* window, wvp width of vertical plate

Morphological terminology follows Courtney et al. (2000) and Szpila and Pape (2007). The term “inter-band area” is introduced for the surface of integument lying between the anterior and posterior spinose bands of particular segments of the larval body (Fig. 1j, k).

Measurements applied for definition of the size of opening of spiracular cavity are illustrated in Fig. 1d, e.

## Results

Key for identification of the third instar larvae of European species of Sarcophagidae of forensic importance

1. Spiracular cavity not developed, posterior spiracles on an only slightly concave spiracular field (Fig. 1a); window in dorsal cornu of cephaloskeleton very short, indistinct (Fig. 1b) . . . *Phylloteles pictipennis* Loew
  Spiracular cavity developed as a deep depression (Fig. 1d, e); window in dorsal cornu of cephaloskeleton long, readily visible (Fig. 1c) . . . 2
2. Entrance of spiracular cavity small, its dimension constitutes at most 0.40 of the width of the anal division in posterior view (Fig. 1d: ‘a/b’) . . . 3
  Entrance of spiracular cavity broad, its dimension constitutes at least 0.45 of the width of the anal division in posterior view (Fig. 1e: ‘a/b’) . . . 4
3. Anterior spiracle with five to seven lobes (Fig. 1f), fully grown post-feeding larva at most 10 mm long . . . *Sarcophila latifrons* (Fallén)
  Remarks. One or more additional species are found in the Mediterranean region, but the taxonomy is unclear and in need of revision (Lehrer 2003; Arnaldos et al. 2004).
  Anterior spiracles with more than seven lobes (Fig. 1g), fully grown post-feeding larva at least 10 mm long . . . *Wohlfahrtia nuba* species group
    Remarks. The most common species are *W. indigens* Villeneuve, *W. nuba* (Wiedemann), *W. trina* (Wiedemann) and *W. villeneuvi* Salem; flies are abundant in desert and semi-desert ecosystems of North Africa, Middle East and Cyprus, southeast zone of European part of Russia.
4. Anterior spiracle broad with lobes arranged in almost straight line (Fig. 1h); dorsal cornu of cephaloskeleton with short window (Fig. 1i): ventral cornu half as long as width of vertical plate (Fig. 1i) . . . *R. pernix* (Harris)
  Anterior spiracle with lobes arranged in an arc (Fig. 3f–l); dorsal cornu of cephaloskeleton with long window (Figs. 1c and 2c, d): ventral cornu as long as width of vertical plate (Figs. 1c and 2c, d) . . . 5

**Fig. 2:**
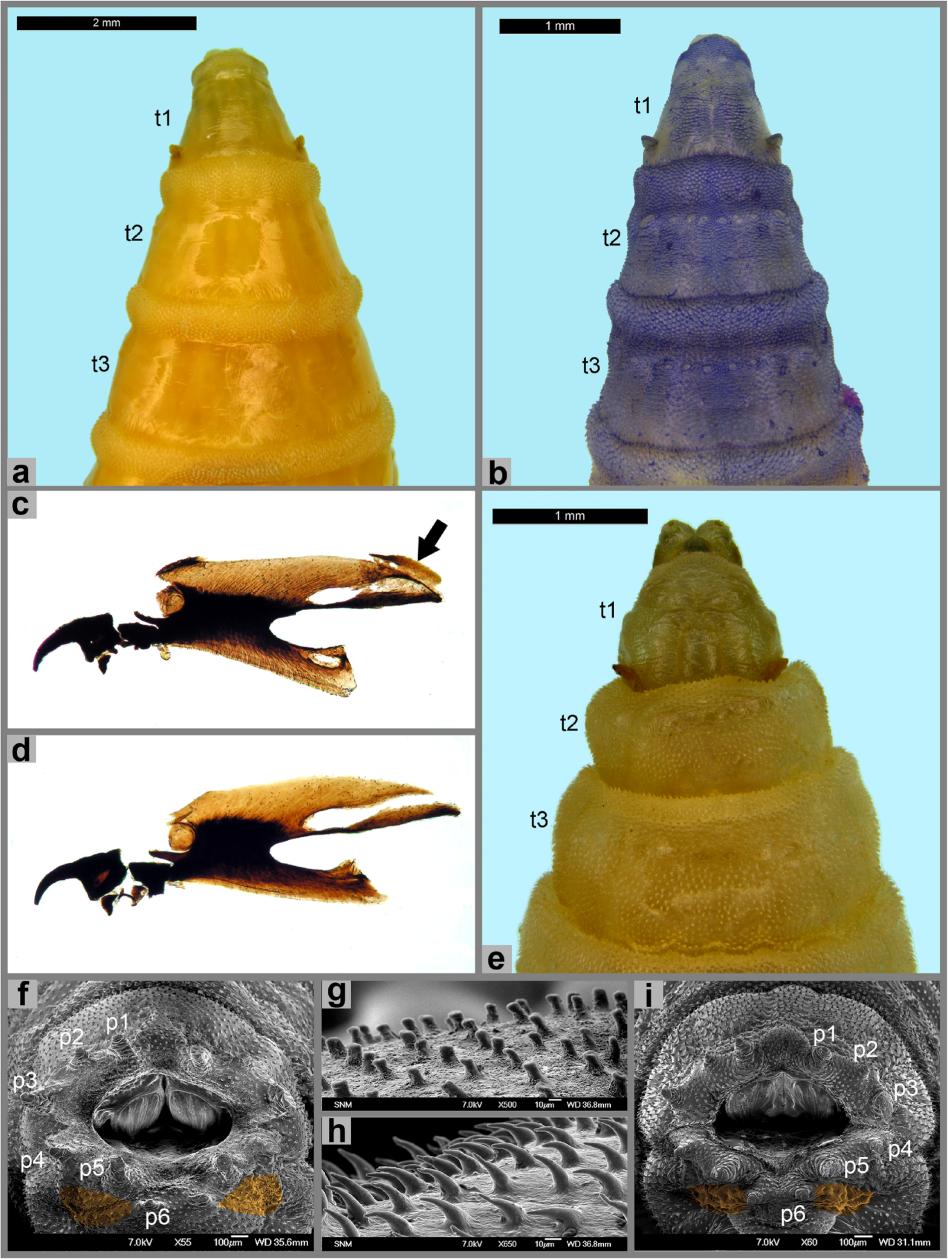
Third instar morphology of Sarcophagidae. **a** Anterior end of body, dorsal view, *Sarcophaga argyrostoma*. **b** Anterior end of body, dorsal view, *S. melanura*. **c** Cephaloskeleton, lateral view, *S. melanura*. **d** Cephaloskeleton, lateral view, *S. africa*. **e** Anterior end of body, dorsal view, *S. africa*. **f** Anal division, posterior view, *S. melanura*. **g** Anal division, spines on dorsal surface, *S. melanura*. **h** Anal division, spines on dorsal surface, *S. africa*. **i** Anal division, posterior view, *S. africa*. Abbreviations: *p1–p6* papillae 1–6, *t1–t3* thoracic segments 1–3
5. Inter-band area with warts/spines on dorsal surface of at least last abdominal segment and anal division (Fig. 1j) . . . 6
  Inter-band area without warts/spines on dorsal surface of abdominal segments and anal division (Fig. 1k) . . . 11
6. Lobes of anterior spiracle arranged in one row (Figs. 1g and 3i, j) . . . 7
  Lobes of anterior spiracle arranged in two-three irregular rows (Fig. 3f–h) . . . 9

**Fig. 3:**
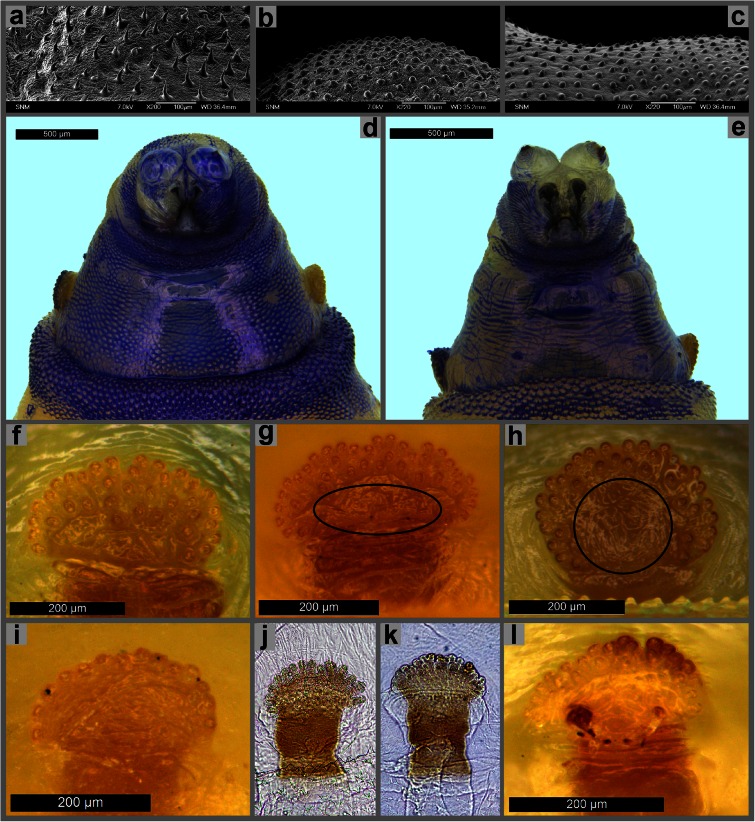
Third instar morphology of Sarcophagidae. **a** Anal division, spines on dorsal surface, *Sarcophaga albiceps*. **b** Anal division, spines on dorsal surface, *S. caerulescens*. **c** Anal division, spines on dorsal surface, *S. similis*. **d** Anterior end of body, ventral view, *S. caerulescens*. **e** Anterior end of body, ventral view, *S. similis*. **f** Anterior spiracle, *S. albiceps*. **g** Anterior spiracle, *S. caerulescens*. **h** Anterior spiracle, *S. similis*. **i** Anterior spiracle, *S. aegyptica*. **j** Anterior spiracle, *S. aegyptica*. **k** Anterior spiracle, *S. variegata*. **l** Anterior spiracle, *S. variegata*
7. At least anterior part of second thoracic segment without spines/warts on inter-band area (Fig. 2a); inter-band area on anal division covered with wart-like spines (Fig. 3b, c) . . . *Sarcophaga* (*Liopygia*) *argyrostoma* (Robineau-Desvoidy)
  Remarks. Two additional species of subgenus *Liopygia* Enderlein are present in the Mediterranean region: *S. crassipalpis* Macquart and *S. cultellata* Pandellé; a preliminary key for the identification of the third instar larvae of European species of subgenus *Liopygia* was recently published by Ubero-Pascal et al. (2015).
  Inter-band area with warts/spines on second and third thoracic and all abdominal segments (Fig. 2b, e); spines of inter-band area on anal division blunt or tapering but never wart-like (Fig. 2g, h) . . . 8
8. Inter-band area dorsally on first thoracic segment with spines/warts (Fig. 2b); entire posterior surface of anal division with warts/spines (Fig. 2f); warts/spines with blunt tip (Fig. 2g); apex of ventral cornu of cephaloskeleton postero-dorsally with an additional strongly sclerotised piece (Fig. 2c) . . . *S.* (*Helicophagella*) *melanura* Meigen
  Inter-band area dorsally on first thoracic segment without spines/warts (Fig. 2e); posterior surface of anal division with spines except lateral to papilla p6 (Fig. 2i); spines with pointed tip (Fig. 2h); apex of ventral cornu of cephaloskeleton simple and without additional strongly sclerotised piece (Fig. 2d) . . . *Sarcophaga* (*Bercaea*) *africa* (Wiedemann)
9. Spines on inter-band area of thoracic and abdominal segments elongated and pointed (Fig. 3a) . . . *Sarcophaga* (*Parasarcophaga*) *albiceps* Meigen
  Spines on inter-band area of thoracic and abdominal segments in form of wart-like, rounded swellings (Fig. 3b, c) . . . 10
10. Inter-band area on ventral surface of the first thoracic segment with warts along posterior margin of segment (Fig. 3d); anterior spiracle oval (Fig. 3g) . . . *Sarcophaga* (*Robineauella*) *caerulescens* Zetterstedt
  Inter-band area on ventral surface of the first thoracic segment without warts/spines (Fig. 3e); anterior spiracle circular (Fig. 3h) . . . *Sarcophaga* (*Pandelleisca*) *similis* Meade
11. Lobes of anterior spiracle arranged in one, sometimes slightly irregular row (Fig. 3i, j) . . . *Sarcophaga* subgenus *Liosarcophaga* Enderlein
  Remarks. The most important species are *S. aegyptica* Salem, *S. dux* Thomson, and *S. tibialis* Macquart—widespread in the Mediterranean region.
  Lobes of anterior spiracle arranged in two or more irregular rows (Fig. 3k, l) . . . *Sarcophaga* (*s.str.*) *carnaria* species group
    Remarks. Facultative or obligatory parasites/predators of earthworms; erroneously identified as typical carrion breeders. The most common species are *S. carnaria* (Linnaeus), *S. lehmanni* Mueller, *S. subvicina* Rohdendorf, and *S. variegata* (Scopoli).

## Discussion

Larvae of necrophagous species of higher Diptera are difficult to identify due to their simplified external morphology and high level of inter-specific similarity. The set of characters usually used in identification keys of third instar larvae is narrow. Recently, Singh et al. (2012) summarised the features most commonly used in the larval taxonomy of Sarcophagidae. They are as follows: the shape of particular elements of the cephaloskeleton, the configuration of anterior spiracles, the shape and distribution of spines in the spinose bands, the form and position of posterior spiracles, and the position and size of papillae around the entrance of the spiracular cavity (Greene 1925; Zimin 1948; Kano et al. 1951; Sanjean 1957; Ishijima 1967; Nandi 1980; Sukontason et al. 2010; Velásquez et al. 2010; Ubero-Pascal et al. 2015). Additionally, Zimin (1948) and subsequently a few other authors, in addition to the standard characters, proposed using the sculpture of the integument for species identification. Since the publication of Nandi (1980), several important papers discussing the taxonomic usefulness of larval characters of higher Diptera have been published (e.g. Erzinçlioğlu 1985; Wallman 2001; Szpila 2010). However, they refer to blowflies, and characters used for identification of the third instar larvae of flesh flies have so far not been critically revised. Conclusions drawn from data in the available literature and studies of original material conducted by the authors have led to the following critical review of the diagnostic importance of selected larval characters at least for the third instar of European species of forensic importance.

### Cephaloskeleton

The most demanding is the analysis of particular parts of the cephaloskeleton using two kinds of microscope slides: flat and concave. The cephaloskeleton is a three-dimensional structure, and squeezing by the cover slip on a flat slide will often produce artefacts or distortions through tilting or compression. Especially prone to erroneous interpretations are the width of the apical part of mouthhooks and the general shape of the intermediate sclerite. Strong squeezing by the cover slip will shift the natural parallel position of both mouthhooks, and in lateral view, the single mouthhook will appear wider by partial exposure of the second mouthhook below. Squeezing of the cephaloskeleton may also result in tilting and compression of the intermediate sclerite with the result that the transverse bridge of the intermediate sclerite is exposed so as to create the impression of a triangular shape of this sclerite in lateral view. Using a flat slide may also cause a problem with reliable assessment of the width of ventral and dorsal cornua. Good examples, where all the problems mentioned here can be traced on the photographs, are found in some recent publications (e.g. Sukontason et al. 2010, fig. 1c; Szpila 2010, fig. 3.3a; Ubero-Pascal et al. 2015, fig. 12a). A solution for reliable analysis of the shape of mouthhooks and dorsal and ventral cornua is the sagittal section of the cephaloskeleton. Unfortunately, the sagittal section results in the destruction of the intermediate sclerite and dorsal bridge (Richet et al. 2011), and for a proper analysis of these sclerites, concave slides must be used or alternative non-invasive clearing methods as proposed by Niederegger et al. (2011). In the larval taxonomy of flesh flies, the level of sclerotisation (pigmentation) of some elements of the cephaloskeleton has also been used (e.g. Ishijima 1967; Nandi 1980). This character seems to be unreliable as it is dependent of larval age as well as the method of preparation and preservation of the material (Erzinçlioğlu 1985). A remark should also be made on the presence of a long posterior incision (window) in the dorsal cornu used as a family-specific character for Sarcophagidae (e.g. Ishijima 1967). This window is short and unclear for observation in third instar larvae of Miltogramminae (Szpila and Pape 2007) and cannot be considered as a diagnostic larval character for the entire family Sarcophagidae.

### Anterior spiracles

Anterior spiracles as well visible external structures are well predisposed to be an important structure for species identification. In the taxonomy of third instar larvae of Sarcophagidae are used the general shape of spiracles, number of spiracular lobes and pattern of arrangement of lobes. All of these characters may be checked on intact third instar larvae using a mid-range stereomicroscope (magnification ×40–×50). The number of spiracular lobes presents some intraspecific variation and often overlaps inter-specifically, which is well known in larvae of Calliphoridae (e.g. Erzinçlioğlu 1985). More useful is the general shape of the spiracle (oval, circular, with straight anterior edge) and especially the arrangement of lobes (one row, multiple rows), which is a character used frequently by former and contemporary authors (Bohart and Gressitt 1951; Kano et al. 1951; Sanjean 1957; Ishijima 1967; Nandi 1980; Sukontason et al. 2010; Velásquez et al. 2010; present paper).

### Shape and distribution of spines (spinulation and integumental sculpture)

Characters relating to spinulation are widely used in the taxonomy of necrophagous Diptera (e.g. Erzinçlioğlu 1985; Wallman 2001; Szpila 2010; Grzywacz et al. 2014), including Sarcophagidae (Zimin 1948; Sanjean 1957; Ishijima 1967; Nandi 1980; Ubero-Pascal et al. 2015). Those most often applied are the distribution and degree of development of spinose bands on the abdominal segments. Spinulation shows a low intraspecific variation and is therefore a very valuable taxonomic character (Erzinçlioğlu 1985; Wallman 2001). However, in Sarcophagidae, the systematic description of the bands may be hampered by the presence of additional small spines/warts on large areas of the central surface of segments (here provided with the term inter-band area). This integumental sculpture vary from long, hair-like spines to low warts, and with many intermediate forms. Distribution of surfaces with various sculpturing on particular segments and the shape of spines/warts show low inter-specific variation and is often a highly diagnostic character for larvae of particular species. The level of development of integumental sculpture was first widely used by Zimin (1948) and Bohart and Gressitt (1951) and also—to a much lesser extent—by Sanjean (1957) and Ishijima (1967). Interestingly, the taxonomic value of this character was overlooked in recent papers documenting the morphology of the Sarcophaginae larvae despite using SEM technique (Sukontason et al. 2010; Singh et al. 2012; Ubero-Pascal et al. 2015). The degree of development of spinose bands and integumental sculpture is easily observed on intact specimens using a standard stereomicroscope, especially if the surface of the larval body is given more topographical contrast using a simple ink marker. A thorough study of the shape of spines needs a more high-powered stereomicroscope as well as preparation of microscope slides or SEM (Szpila and Pape 2007).

### Spiracular cavity and accompanying structures

The presence of a posterior spiracular cavity is widely used as a family-specific character of the Sarcophagidae. However, as for the window of the dorsal cornu in the cephaloskeleton, this morphological modification is not well developed in third instar larvae of necrophagous species representing the subfamily Miltogramminae (Szpila and Pape 2007; Szpila et al. 2010). Third instar larvae of Miltogramminae (at least in the common West Palaearctic *P. pictipennis* and the New World *Eumacronychia persolla* Reinhard and *Eumacronychia sternalis* Allen) possess only a slightly subducted spiracular field with broad exposure of the posterior spiracles. Using some popular family-level keys for the identification of larvae of Diptera inhabiting carrion (e.g. Ishijima 1967; Smith 1986) may therefore lead to a misidentification of larvae of Miltogramminae with assignment to families Muscidae or Calliphoridae. Within the family Sarcophagidae, it is difficult to distinguish between third instar larvae of necrophagous Paramacronychiinae and Sarcophaginae. It is not a serious obstacle for Central and North European species where necrophagous Paramacronychiinae are represented only by the single species *S. latifrons*. In the Mediterranean region and southeast Europe, a few additional species of *Sarcophila* Rondani are present, but also several species of the genus *Wohlfahrtia* Brauer & Bergenstamm, which frequently inhabit carrion in dry habitats (e.g. Tantawi et al. 1996). Contrary to larvae of *Sarcophila*, large third instar larvae of *Wohlfahrtia* are easily misidentified as larvae of *Sarcophaga* with undeveloped integumental sculpture, especially representing the subgenus *Liosarcophaga*. A helpful character in this connection is the size of the entrance to the spiracular cavity, already mentioned by Greene (1925). In fully grown third instar larvae of necrophagous Paramacronychiinae, this entrance is distinctly smaller than for the Sarcophaginae. The size and position of the papillae situated around the spiracular cavity is a frequently used character in larval taxonomy of higher Diptera (e.g. Szpila 2010). In Sarcophagidae, these papillae have the form of fleshy processes, and keys have in particular been using information about the three pairs of papillae situated dorsally and dorso-laterally (Greene 1925; Zimin 1948; Kano et al. 1951; Sanjean 1957; Velásquez et al. 2010). With the exception of some North American species (Greene 1925; Sanjean 1957), the taxonomic value of the size of these papillae seems to be of low reliability. Authors have rejected the character proposed by Zimin for distinguishing larvae of *S. africa* and *S. melanura*—the relative size of papillae p1 to p3. Differences in size are blurred and difficult to assess (Fig. 2f, i). Velásquez et al. (2010) proposed in their key another character related to the morphology of the spiracular cavity—convexity of ventral surface of the spiracular cavity. This convexity, present exclusively in the third instar larva of *R. pernix*, partially screens the posterior spiracles which are not fully visible in a posterior view of the spiracular cavity. However, the presence of this bulge may be related to the methods of killing, handling and preservation of larval material and should rather not be treated as a strong taxonomic character.

### Posterior spiracles

Information on the morphology of posterior spiracles is frequently used in keys for the identification of larvae of flesh flies. Detailed characters are usually relating to the level of sclerotisation and the shape of the peritreme or the shape of the inner spiracular slit (Bohart and Gressitt 1951; Sanjean 1957; Ishijima 1967; Nandi 1980; Sukontason et al. 2010; Velásquez et al. 2010; Ubero-Pascal et al. 2015). Our extensive studies of original larval material point to low taxonomic reliability of these characters. The degree of sclerotisation of the peritreme, expressed as the degree of development of the ventral arch and inner projections of the peritreme between the spiracular slits, shows clear intraspecific variation. Larvae of *S. caerulescens* in the key of Ishijima (1967, as “*Robineauella scoparia*”) are characterised by absence of a sclerotised ventral arch. Sanjean (1957) and Richet et al. (2011) present illustrations of the posterior spiracles of the same species with a developed ventral arch. Larvae of *S. caerulescens* checked for the present paper possessed posterior spiracles with various levels of sclerotisation, which seemed to be correlated to larval age. Additionally, subtle differences in level of sclerotisation of the peritreme may be affected by methods of preparation of material (Erzinçlioğlu 1985). However, it is important to notice that a total absence of sclerotisation of the ventral arch of the peritreme was observed in all checked larvae of Paramacronychiinae and of the *S. carnaria* species group, which in case of the latter taxon finds confirmation in available literature data (Draber-Mońko 1973; Richet et al. 2011). Velásquez et al. (2010) suggested that the inner slit of the posterior spiracle of *S. africa* is straight as compared to other larvae of flesh flies, where this slit is slightly curved. A review of literature data (Sanjean 1967; Richet et al. 2011) and original material shows that the inner slit of the posterior spiracle of *S. africa* may also be more or less curved, which means that this character cannot be considered as reliable for species identification.
